# COVID‐19 Mobile Brain Health

**DOI:** 10.1002/alz.088216

**Published:** 2025-01-03

**Authors:** Ashita S. Gurnani, Stephanie Li, Phillip H Hwang

**Affiliations:** ^1^ Framingham Heart Study, Boston University Chobanian & Avedisian School of Medicine, Boston, MA USA; ^2^ Boston University Chobanian & Avedisian School of Medicine, Boston, MA USA; ^3^ The Framingham Heart Study, Framingham, MA USA; ^4^ Boston University School of Public Heatlh, Boston, MA USA

## Abstract

**Background:**

The long‐term neurological impact of the SARS‐CoV‐2 virus is unknown and it remains to be seen whether it would create a surge in cases of dementia and cognitive decline years later, which is already a global public health challenge. Our group has previously shown that participants cognitive functioning as measured via mobile‐based assessments using smartphone‐based cognitive tests did not differ based on their COVID status. The goal of the present study was to examine participants longitudinal cognitive performance with the hypothesis that participants with a previous COVID‐19 diagnosis (COVID+) will have worse cognitive performance over time than those without COVID‐19 (COVID‐).

**Method:**

Participants (n = 45) with self‐reported positive (n = 25) or negative COVID‐19 (n = 20) statuses based on polymerase chain reaction or antigen testing were recruited from the Boston area. Inclusion criteria included access to a smartphone with an Android or iOS operation system and to internet connectivity, along with proficiency in English. Cognitive performance was measured using Defense Automated Neurobehavioral Assessment (DANA) from AnthroTronix. Participants were asked to complete DANA monthly over six months. Generalized estimating equations (GEE) were used to compare longitudinal cognitive performance among those with and without COVID‐19.

**Result:**

The sample was, on average, 53 years of age and comprised primarily of COVID+ (56%), female (78%), and Caucasian (50%) participants that were generally well educated (42% with at least a bachelor’s degree), and had ≥1 COVID vaccination (95%). Participants completed an average of 5.5 DANA assessments over the six‐month period. Results indicated that after adjusting for age, sex, and education, COVID+ group had significantly greater decline in performance on the Code Substitution (*β* = ‐2.14, 95% CI = ‐4.05 – ‐0.24, *p* = 0.028) and Match to Sample tasks (*β* = ‐2.01, 95% CI = ‐3.80 – ‐0.25, *p* = 0.026) in comparison to the COVID‐ group.

**Conclusion:**

Results indicate that while cognitive performance at baseline does not differ based on COVID status, having COVID may impact working memory and sensory processing abilities over time. Future directions include examining the impact of COVID disease severity and reinfection on cognition.

